# Discontinuity-Induced Dynamics in the Conductance-Based Adaptive Exponential Integrate-and-Fire Model

**DOI:** 10.1007/s11538-024-01384-z

**Published:** 2024-11-14

**Authors:** Mathieu Desroches, Piotr Kowalczyk, Serafim Rodrigues

**Affiliations:** 1 MathNeuro Project-Team, Inria Branch of the University of Montpellier, Montpellier, France; 2 MCEN Team, BCAM - Basque Center for Applied Mathematics, Bilbao, Spain; 3 Department of Mathematics, Wrocław University of Science and Technology, Wrocław, Poland; 4 Ikerbasque, The Basque Science Foundation, Bilbao, Spain

**Keywords:** Neuronal model, Discontinuity-induced bifurcation, Integrate-and-fire models, Slow-fast dynamics, Canards

## Abstract

In this article, we present a computational study of the Conductance-Based Adaptive Exponential (CAdEx) integrate-and-fire neuronal model, focusing on its multiple timescale nature, and on how it shapes its main dynamical regimes. In particular, we show that the spiking and so-called *delayed bursting* regimes of the model are triggered by *discontinuity-induced bifurcations* that are directly related to the multiple-timescale aspect of the model, and are mediated by canard solutions. By means of a numerical bifurcation analysis of the model, using the software package coco, we can precisely describe the mechanisms behind these dynamical scenarios. Spike-increment transitions are revealed. These transitions are accompanied by a fold and a period-doubling bifurcation, and are organised in parameter space along an *isola* periodic solutions with resets. Finally, we also unveil the presence of a homoclinic bifurcation terminating a canard explosion which, together with the presence of resets, organises the *delayed bursting* regime of the model.

## Introduction

In the present work, we focus on investigating the dynamics of so-called Conductance-Based Adaptive Exponential Integrate-and-Fire Model of a neuron (CAdEx). This model was introduced in Górski et al. (2021), specifically to reproduce a range of so-called firing patterns mimicking electrical properties of neurons. As mentioned in Górski et al. (2021), this model may be thought of as a *minimal biophysical neuron model*. That is, a model with enough complexity to capture the essential firing patterns of neurons, but sufficiently simplified to allow for a large-scale neural network computations. From the mathematical neuroscience viewpoint, of particular interest is the ability to capture a specific behaviour of neural systems termed as *spiking* and *bursting*. There are many low-dimensional models which capture these behaviours; see for instance FitzHugh (1961), Morris and Lecar (1981), Izhikevich (2003), Brette and Gerstner (2005), Naud et al. (2008), Coombes et al. (2012).

The CAdEx belongs to a class of low-dimensional models with two dynamic variables, one of which corresponds to the voltage *V* across the neural membrane, and the other to a conductance-based adaptation $g_A$; see system (1). Additionally, there is a reset of the voltage variable after a certain threshold value is reached, concomitantly with an increment of the conductance variable; see (2). There are two features of the current model which should be stressed. Firstly, this model has two timescales, even though there is no explicit timescale parameter. Namely, as often in neuron membrane models, the voltage variable evolves on a much faster timescale than the conductance variable. Secondly, this is a switched model. That is, as mentioned above, when the voltage variable reaches some (fixed) threshold value, it is then reset to a lower (fixed) value and the conductance is incremented by a set amount. This mimics spiking activity without actually computing spikes, and it is the modeling strategy underpinning all integrate-and-fire models like the CAdEx. In Górski et al. (2021), the authors perform a local bifurcation analysis of the model in the subthreshold region where, depending on the system’s parameters, they identify multistability, saddle-node bifurcations and Andronov-Hopf bifurcations. Then, they describe six distinct dynamical scenarios involving rest states and different types of spiking behviour, which they consider important from the neural modelling perspective. These are listed in Table 1 in the Appendix. They do not explain, however, how, after the transient, the number of spikes, and hence the frequency of oscillations, change under parameter variation. It is this aspect of the dynamics that we explain numerically in the current work, by linking it with a Discontinuity Induced Bifurcation (DIB) that we explained analytically in Desroches et al. (2021), and for which numerical evidence can be given in the present model. The DIB reported and analysed in Desroches et al. (2021) in a simpler piecewise-linear version of the CAdEx model, results in a spike-increment, which, in turn, is the result of an interaction between the repelling branch of the so-called *critical manifold*, that is, the *V*-nullcline (since *V* is a fast variable), and the reset mechanism. Namely, when the reset of the trajectory is “close enough” to the repelling branch of the critical manifold, the solution follows this branch for some time, hence displaying a *canard segment* (Dumortier and Roussarie 1996; Krupa and Szmolyan 2001). These specific solutions exist within very small intervals in parameter space and, in the present context, they connect families of bursting solutions with different numbers of spikes per burst.

Thus, the main objective of the present work is to showcase such reset-induced canard dynamics and how they trigger and affect some of the spiking and bursting patterns reported in Górski et al. (2021). We provide numerical evidence for these *spike-increment scenarios*. We then show numerical evidence of a homoclinic bifurcation, the effects of canard segments on the stability of periodic cycles, and we also present bifurcation diagrams of periodic solutions with resets. Finally, we numerically describe the dynamics triggered by the presence of a fold point on the critical manifold, and canard cycles present in the subthreshold regime.

The results presented here are relevant to the analysis and numerical simulations of nonsmooth dynamical systems. In particular, the results relate to issues which are relevant to the stability of periodic solutions in nonsmooth systems. To compute the stability of periodic solutions in systems with switchings, or resets, monodromy matrices (Seydel 1994) have to be composed with so-called saltation matrices, or matrices which are linearisation terms of so-called discontinuity mappings, taking into account the presence of switchings, or resets (Di Bernardo et al. 2008; Leine and Nijmeijer 2004). In the standard theory of nonsmooth systems, the variation of the nontrivial Floquet multipliers of periodic solutions with respect to parameter variations may have jumps, due to, for example, grazing bifurcations (Kowalczyk and Piiroinen 2007; Nordmark 2001). In this case, if a grazing scenario takes place, for example in an impact oscillator, one multiplier tends to infinity (Nordmark 1991), if the limit is taken from the “impacting side”. However, further away from a DIB, the variation of the nontrivial Floquet multipliers is continuous and not abrupt (Adolfsson et al. 2001; Di Bernardo et al. 2008). In the current model, we observe large variations of the nontrivial Floquet multiplier on an exponentially small interval of parameter variation. This is clearly the effect of the slow-fast nature of the problem, and in particular, the instability is triggered by the presence of canard segments making up part of the periodic cycles. This effect is classical in planar smooth slow-fast systems, and it has been analysed in, e.g., Dumortier and Roussarie (1996) and in Krupa and Szmolyan (2001). This is the first time that such a behaviour of the nontrivial Floquet multiplier with respect to parameter variation is reported in the context of nonsmooth dynamical systems.

In view of presenting these results, the rest of the article is organised as follows. In Sect. 2, the equations describing the model are introduced and the slow-fast structure of the model is described. We then define limit cycles containing canard segments and give numerical evidence for spike-increment scenarios and isolas in the CAdEx model In Sect. 3. In Sect. 4, we present an effect of canard segments on the character of limit cycle solutions with resets. In particular, we show the effects on the stability as well as on the sensitivity of the limit cycles with respect to parameter variations. We also present and discuss numerical results of a homoclinic bifurcation of a 5-reset cycle. In Sect. 5, we discuss the effect of a fold point on the presence of canard cycles in subthreshold dynamics, in conjunction with a homoclinic bifurcation. Finally, In Sect. 6 we first discuss our results by referring back to neural modelling, and then present conclusions which, we believe, are of interest to the computational neuroscience community, as well as list various analytical and computational problems related to systems with discontinuous nonlinearities.

## The CAdEx Model

The system of interest, as already mentioned, contains two state variables, which are named *V* and $g_A$, and denote the voltage and a conductance-based adaptation, respectively. Additionally, a reset of the voltage variable is applied when a certain fixed threshold value of the voltage, say $V_D$, is reached. The reset also changes the conductance, which is incremented by a discrete positive amount $\delta g_A$. It is important to notice the geometry of the $g_A$-nullcline, which is sigmoidal. This modification from the AdExp model, as explained in Górski et al. (2021), allows for the value of conductance after a train of spikes to limit the current in such a way that it is not unrealistically strong. Next, we introduce the model’s equations.

### Model’s Equations

The subthreshold dynamics of the CAdEx model are organised by the following set of differential equations:

1 $$\begin{aligned} \begin{aligned} C_m \frac{dV}{dt}&= g_L(E_L - V) + g_L\Delta _T\text{ exp }\left( \frac{V - V_T}{\Delta _T} \right) + g_A(E_A - V) + I_s\\ \tau _A \frac{d g_A}{dt}&= \frac{\bar{g}_A}{1 + \text{ exp }\left( \frac{V_A - V}{\Delta _A} \right) } - g_A. \end{aligned} \end{aligned}$$

When the voltage reaches its threshold value, a reset function, defined as:

2 $$\begin{aligned} \text{ if }\quad V \ge V_D \quad \text{ then }\quad {\left\{ \begin{array}{ll}V\mapsto V_R, g_A \mapsto g_A + \delta g_A, \end{array}\right. } \end{aligned}$$

is activated. Effectively, taking into account the relative timescales of state variables, as well as the instantaneous reset/increment, one may view such a model system as a three-dimensional dynamical system with two fast and one slow variable; see Sect. 2.2 below. Before we discuss the slow-fast nature of the model, following Górski et al. (2021) we explain the physical meaning of Eq. (2) parameters. Consider the voltage equation in (1). Parameter $C_m$[pF] describes the neuronal membrane capacitance; $g_L$[nS] is the leak conductance; $E_L$[mV] is the leak reversal potential; $E_A$[mV] is the reversal potential of the adaptation conductance; $I_s$[pA] is the input current; $V_T$[mV] is the spike threshold and $\Delta _T$[mV] is the slope of the spike initiation. Note that when the voltage exceeds $V_T$[mV], the exponential term starts dominating the voltage equation and a spike is initiated. We now turn to the conductance equation in (1). Parameter $\tau _A$[mS] is the time constant of adaptation; $\bar{g}_A$[nS] is the maximal subthreshold adaptation conductance; $V_A$[mV] is the subthreshold adaptation activation voltage; $\Delta _A$[mV] is the slope of subthreshold adaptation; $V_D$[mV] is the saturation limit of the voltage, meaning that when it is reached the voltage is reset to the potential $V_R$[mV], and the conductance is incremented by some positive amount $\delta g_A$[nS]. The $g_A$-nullcline, which is a sigmoidal adaptation function, is non-negative, while the *V*-nullcline is parabolically shaped.

### Slow–Fast Structure of the Equations

**Fig. 1:**
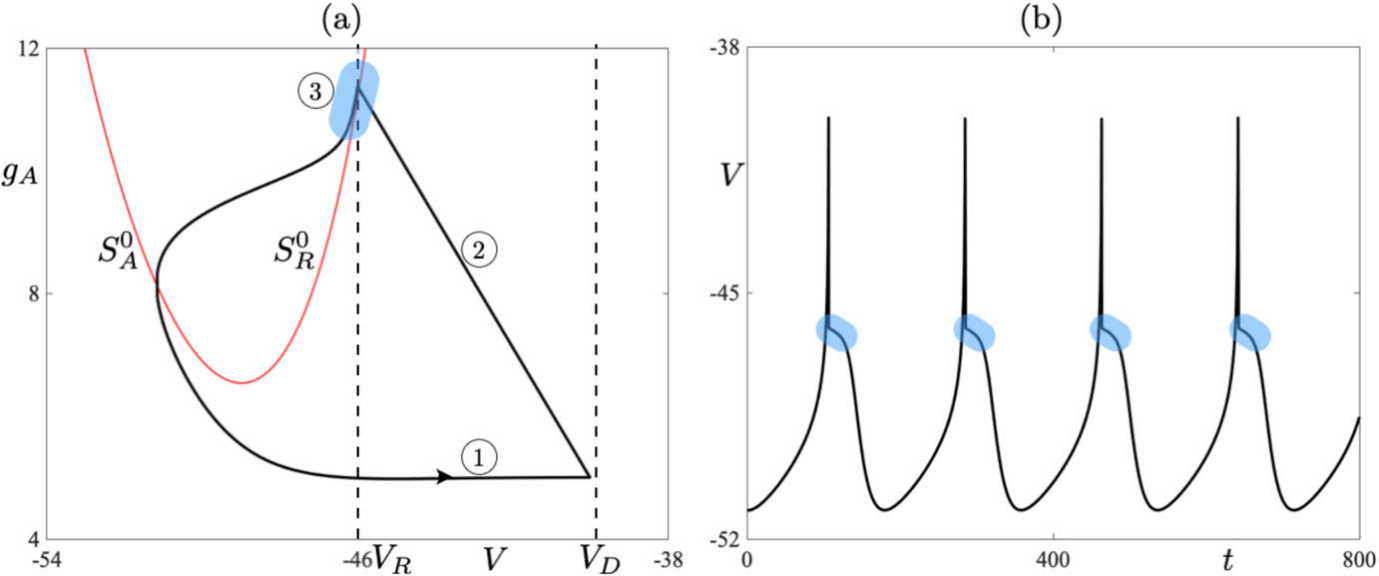
One-reset canard cycle in the CAdEx model (1)-(2), shown in the phase plane (**a**) and in time for the variable *V* (**b**). The phase-plane projection also shows the attracting, $S^0_A$, and the repulsive, $S^0_R$, parts of the quadratic-shaped critical manifold, as well as, the threshold line $\{V=V_D\}$ and the reset line $\{V=V_R\}$. The cycle contains a fast attracting segment (label 1), an instantaneous reset segment (label 2) and a slow repulsive segment (label 3, also highlighted by a colored region). Parameter values are those of the *bursting* case (see Table 1) except for: $\delta g_A=6.365$ and $I_s=129$ (Color figure online)

We now discuss the structure of the CAdEx system (1). In particular, we choose to focus on timescale separation between the evolution of the state variables *V* and $g_A$, and what it entails. From the nondimensionalization analysis (see Appendix A), it follows that the *V* variable evolves on a fast timescale in comparison with that of the $g_A$ variable. For this reason, we will term the *V* variable as *fast* and the $g_A$ as *slow*, and the timescale separation between the two is measured via the existence of a small parameter $\varepsilon$, which can be defined as the ratio of $C_m$ over $\tau _Ag_L$. Given the geometry of the *V*-nullcline, the aforementioned timescale separation is crucial insofar as it allows for the existence of *slow manifolds* (*attracting* ones and *repelling* ones), as well as, so-called *canard solutions* (Dumortier and Roussarie 1996; Krupa and Szmolyan 2001); see Sect. 3.1 for further details. Considering the dimensional quantities used in system (1), the timescale separation of the evolution between *V* and $g_A$ is at least on the order of 10 depending on the particular case, that is, $\varepsilon$ is on the order of $10^{-1}$ or smaller when nondimensional quantities are considered for comparison purposes. See the Appendix for further details. Therefore, with some abuse of notation, since we express our model in terms of the dimensional variables, we rewrite system (1) in the general form of a slow-fast dynamical system as:

3 $$\begin{aligned} \begin{aligned} \varepsilon \frac{dV}{dt}&= f(V,{g_A}), \\ \frac{d{g_A}}{dt}&= h(V{g_A}), \end{aligned} \end{aligned}$$

where $\varepsilon = O(10^{-1})$, or smaller, and *f*, *h* are nonlinear functions appropriately scaled from the right-hand side of system (1). We will only highlight elements of such systems that are pertinent to our investigations. Having in mind system (3), we can see that, away from the *V*-nullcline, *V* evolves much faster than $g_A$. Combining such an evolution with resets (2) may lead to periodic solutions with resets, which we define in the next section. We now define:

4 $$\begin{aligned} S^0 = \left\{ (V,g_A) \in (-\infty ,\, V_D]\times \mathbb {R}\, :\, f(V,g_A) =0\right\} \end{aligned}$$

to be the *critical manifold* of system (3). It is the union of its attracting and repelling subsets, namely

5 $$\begin{aligned} \begin{aligned} S^{0}_{A}&= \left\{ (V,g_A) \in S^0\, :\, f(V,g_A)_{V} < 0\right\} ,\\ S^{0}_{R}&= \left\{ (V,g_A) \in S^0\, :\, f(V,g_A)_{V} > 0\right\} , \end{aligned} \end{aligned}$$

where the subscript $_{V}$ denotes the partial derivative with respect to *V*. Hence, trajectories are typically attracted to $S^{0}_{A}$ while they are typically repelled from $S^{0}_{R}$. Having highlighted the slow-fast structure of the system, in the next section we define *n*-reset periodic cycles and propose a special focus on cycles that contain a segment staying close to $S^{0}_{R}$ for a long time, even though it is repelling. Such segments qualify as canard segments. Specifically, we consider trajectories containing a fast attracting segment that corresponds to the initiation of the upstroke of the spike, the rise towards the threshold $V_D$, which is followed by a fast (in fact, instantaneous) segment that corresponds to the reset. Finally, this is followed by a slow repelling segment along a repelling slow manifold. The combination of these three segments can be termed a *jump-on canard*; see, e.g., Dumortier and Roussarie (2007). These canard segments play a special role in phase space, as we shall see next. They are illustrated, within a one-reset limit cycle solution of the CAdEx system, in Fig. 1.

## Bifurcations and Discontinuity-Induced Dynamic Transitions

### Canard Cycles

We now define precisely periodic cycles with resets as invariant solutions of system (1)-(2). The definitions below follow those first introduced in Desroches et al. (2021), and we repeat them here for the clarity of exposition. We first define limit cycles with *n* resets, for some integer $n\ge 1$, which we will term *n*-*reset cycles*. Let us also denote by $\phi$ an evolution operator of system (1) for $t \ge 0$ with reset condition (2). This is a forward-time composition of flows and resets. Operator $\phi$ satisfies the standard two properties of evolution operators. Namely: $\phi ^0 = \text{ id }$, where $\text{ id }$ denotes the identity, and $\phi ^{t + s}=\phi ^{t}\circ \phi ^{s}$, where times *t* and *s* are both positive and the symbol $\circ$ denotes composition. It is crucial to note that the evolution operator $\phi$ includes resets, which act in zero time, and their order of occurrence is strictly determined given an initial condition $(V_0,g_0)$.

#### Definition 1

An *n*-*reset periodic cycle* *L*(*V*, *g*, *n*), where *n* is a positive integer, is a subset of the phase space of system (1) such that each point $P_0 = (V_0,g_0)\in L$ satisfies $\phi (V_0,g_0, t) = \phi (V_0,g_0, t + T)\in L$ for some $T > 0$ and any $t\ge 0$. Moreover, there are *n* time instants when system (1) reaches the threshold value $V = V_{\textrm{D}}$ for $t\in (0, \, T]$, starting an evolution at any point $(V_0,g_0)\in L$.

#### Definition 2

An *n*-*reset limit cycle* *L*(*V*, *g*, *n*) (or just *n*-*reset cycle*), where *n* is a positive integer, is a subset of the phase space of system (1) such that *L* is an *n*-reset periodic cycle and a limit set of system (1).

#### Remark 1

Given that the reset is instantaneous, in Definition 1 we have used the time interval in which we count the time instances of resets for $t\in (0, \, T]$. This ensures that if the initial $V_0$ is equal to $V_{\textrm{D}}$, then we do not count it as reaching the threshold twice. Note that if we chose $t\in [0, \, T)$ then if $V_0 = V_{\textrm{R}}$ we might miss out one reset, and hence the latter choice of the time interval is not used in Definition 1.

#### Remark 2

Since we consider only positive times, an *n*-reset cycle according to Definition 2 would imply an attractor since only $\omega$-limit sets would be possible in the system. However, unstable *n*-reset periodic cycles are also possible in system (1). Thus, an *n*-reset limit cycle in the text will refer either to a stable or unstable *n*-reset periodic cycle even though, formally, only $\omega$-limit sets may be defined.

**Fig. 2:**
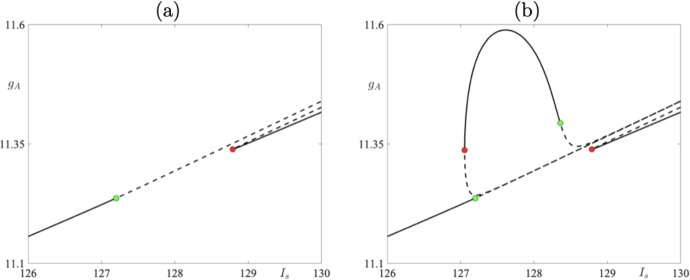
**a** A one-parameter bifurcation diagram depicting periodic cycles with resets. The solid curve denotes stable 7- and 8- spike periodic cycles, and the dashed curve denotes unstable 7 and 8-spike periodic cycles. The green dot corresponds to a period-doubling bifurcation of a 7-spike cycle and the red dot to a fold bifurcation of an 8-spike cycle. **b** Same as in the left panel with an addition of 9-spike periodic cycles lying on an isola. Notice the existence of stable 9-spike solutions between 7- and 8-spike cycles. The color dots on the isola denote fold and period-doubling bifurcations of a 9-spike periodic cycle (Color figure online)

In the following sections, we will primarily focus on the effect of canard cycles on the dynamics. We will not give a precise definition of a canard cycle here, but rather we will highlight a feature which distinguishes such a cycle from a generic *n*-reset cycle that may occur in our model system. In the present case, a canard cycle is a cycle containing a segment of trajectory making up an *n*-reset periodic cycle which closely follows, for some positive time *t*, the repelling part of $S^0$, namely $S^{0}_{R}$; see again Fig. 1. It is worth noting here that such periodic cycles may be either stable or unstable, which depends on the balance between the duration of canard segments and segments of evolution characterised by contraction, which make up a given canard cycle.

### DIB Scenario example: Spike Increment $7 \leftrightarrow 9 \leftrightarrow 8$

We now present numerical investigations of system (1)-(2) using the Matlab toolbox coco.This numerical tool is designed to perform automated bifurcation analysis of systems with discontinuous nonlinearities and resets. In particular, it allows one to construct a continuation problem which provides information on the stability of limit cycle solutions. This is done by numerical computations of so-called discontinuity matrices, which capture the effects of switchings and resets, which are combined with monodromy matrices for segments of trajectories making up a limit cycle solution which are continued in some parameter or parameters; see Dankowicz and Schilder (2013) for details on this numerical continuation package.

**Fig. 3:**
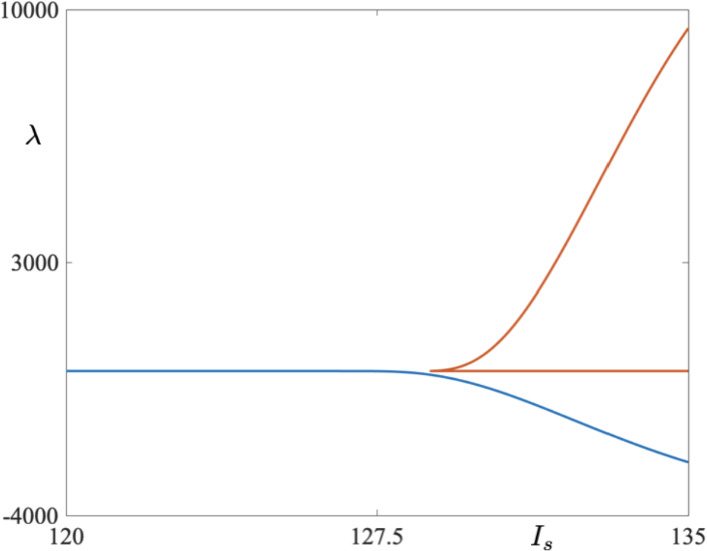
A non-trivial Floquet multiplier corresponding to a one-parameter orbit diagram depicted in Fig. 2 for two families of 8-reset periodic cycles and a family of 7-reset cycles for $I_s \in [120,\, 135]$

**Fig. 4:**
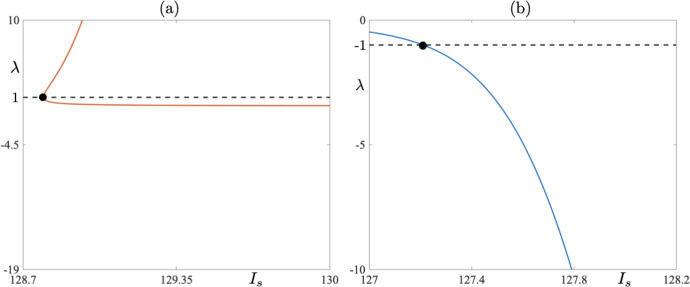
**a** A non-trivial Floquet multiplier corresponding to a one-parameter bifurcation diagram for the family of 8-reset periodic cycles for $I_s\approx 128.8$ where the 8-reset cycles undergo a saddle-node bifurcation. **b** A non-trivial Floquet multiplier corresponding to a one-parameter bifurcation diagram for the family of 7-reset periodic cycles for $I_s\approx 127.2$, which is in the neighbourhood of the $I_s$-value where the 7-reset cycle undergoes a period-doubling bifurcation and loses stability (Color figure online)

To be able to understand how the system’s bifurcation structure and its dynamics change under parameter variation, we focus on a one-parameter bifurcation diagram (with respect to parameter $I_s$) for an 8-reset cycle starting at $I_s = 130$ and then decreasing $I_s$ to $I_s = 126$. Other parameter values are those of the *adaptive spiking* case (see Table 1). We will represent the results in two bifurcation diagrams, one not including an isola structure of 9-reset cycles (Fig. 2a) and the second one including the isola structure of 9-reset cycles (Fig. 2b).

**Fig. 5:**
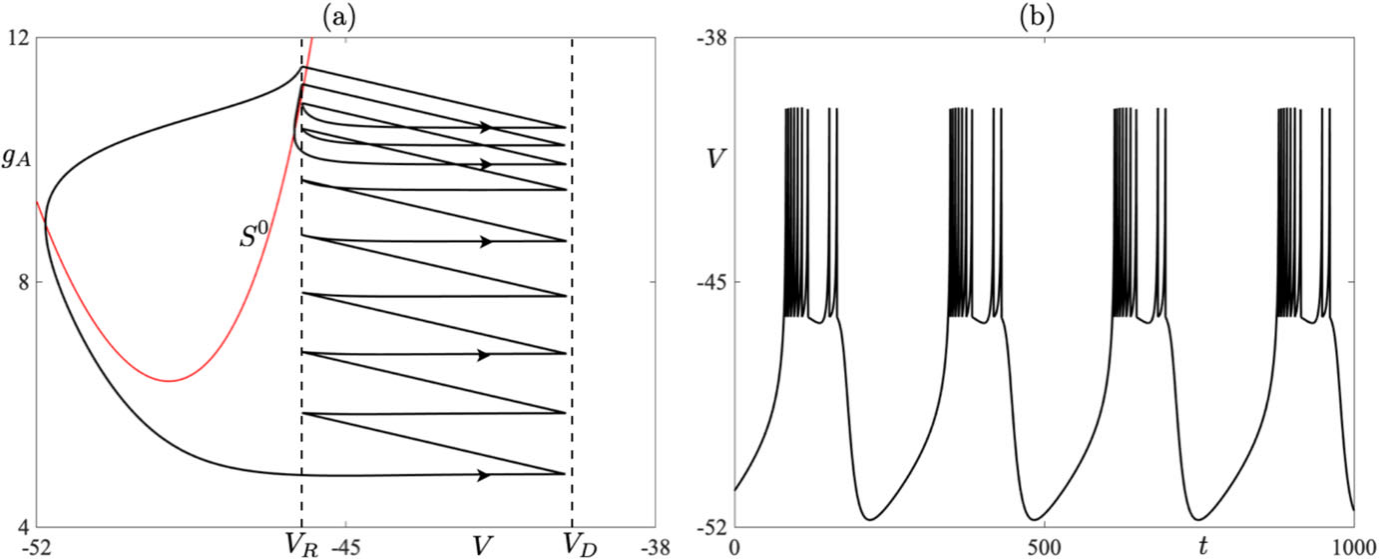
A periodic cycle with 9 resets for $I_s = 127.2$: phase-plane projection (**a**) and membrane potential time profile (**b**). Simulations have been made for the parameter set corresponding to *bursting*; see Table 1 (Color figure online)

**Fig. 6:**
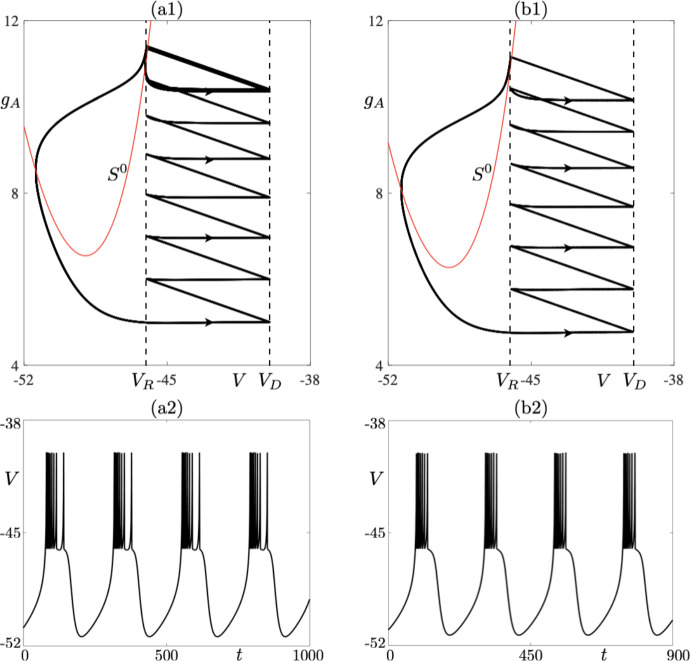
**a1**–**b1**) A periodic cycle with 8 resets for $I_s = 129$: phase-plane projection (**a1**) and membrane potential time profile (**a2**). **b1**–**b2** A periodic cycle with 7 resets for $I_s = 126$: phase-plane projection (**b1**) and membrane potential time profile (**b2**). Both simulations have been made for the parameter set corresponding to *bursting*; see Table 1 (Color figure online)

Consider first Fig. 2. On the *y*-axis we denote the maximum value of $g_A$ variable on the limit cycle at the moment right after the voltage reset takes place, while the *x*-axis represents the corresponding value of parameter $I_s$. Starting from $I_s = 130$, we detect two 8-reset limit cycles — the stable and unstable one (solid curve for the stable ones and dashed curve for unstable ones in Fig. 2). One limit cycle is characterised by a positive nontrivial Floquet multiplier whose modulus is less than one. The other cycle is strongly unstable with the value of the nontrivial Floquet multiplier above 3000 for $I_s = 130$ (see Fig. 3). Decreasing $I_s$ leads to a situation when the two limit cycles collide in a fold bifurcation at $I_s\approx 128.8$; see Fig. 2a for a bifurcation diagram, and Fig. 4a where the values of the Floquet multiplier are depicted. The Floquet multiplier of the colliding cycle is marked in Fig. 4a by a black dot and it corresponds to the red dot in the bifurcation diagrams in Fig. 2a. We can see that the multiplier is equal to 1. Decreasing $I_s$ further causes a jump to a stable 9-reset cycle, depicted in Fig. 2b. A representative cycle of this type, for $I_s = 127.2$, is depicted in Fig. 5. Note on this cycle a trajectory segment that lies in a close proximity of the repelling part of the critical manifold (the red *V*-nullcline), that is, a canard segment. This implies that a fold bifurcation and the following spike loss/increment is driven by the presence of canard orbits. The green dot in the bifurcation diagrams in Fig. 2b, corresponding to 9-reset cycles, denotes period-doubling bifurcation. Further decrease of $I_s$ parameter triggers a jump from a 9-reset cycle to a stable 7-reset cycle at $I_s\approx 127.0538$. We observe a period-doubling bifurcation of a 7-reset cycle at $I_s\approx 127.2$. This 7-reset cycle is stable for $I_s < 127.2$; see the solid curve in the bifurcation diagram in Fig. 2 and the corresponding curve of Floquet multipliers, traced in blue in Fig. 4b. We should indicate here the co-existence of unstable 7- and 8-reset cycles for $I_s > 128.8$; our numerics show that the two unstable cycles co-exist for $I_s$ up to $I_s = 150$, with all other parameters fixed. This co-existence is shown in the bifurcation diagrams in Fig.  2 for $I_s$ up to $I_s = 130$ only, for the clarity of graphical representation. Representative stable limit cycle solutions for three different values of parameter $I_s(k)$, where *k* indicates the number of resets at a given value of $I_s$, with $I_s = I_s^j(k)$ ($j = 1,\,2,\,3)$ and such that $I_s^1(7)< I_s^2(9) < I_s^3(8)$, are depicted in Figs. 5 and 6, both showing the cycles in the phase plane as well as their *V* time profile. In each case, note the presence of a canard segment on the cycle. This scenario is a spike-adding DIB, as discussed in Desroches et al. (2021). That is, under a variation of the bifurcation parameter, say $\mu$ (in our specific case $\mu = I_s$), we observe a transition between a stable 7-reset cycle and a stable $(7+1)$-reset cycle via an intermediary stable invariant set, which in our case is a 9-reset cycle.

We may thus, symbolically, describe the spike increment/loss that we observe in system (1) as a $7 \leftrightarrow 9 \leftrightarrow 8$ transition. There are two additional points which we should make here. Firstly, there may be other invariant sets existing in the parameter interval where both the 7- and 8-reset cycles are unstable, which we do not mention here. Secondly, the scenario described here is driven by a combination of an interaction between canard segments and the reset. In particular, at the bifurcation, there is a canard segment which originates at the reset line, and there exist a nearby parameter value when the canard segment lies to the left, and for some other nearby value, to the right of the reset line; see Desroches et al. (2021) for further details.

### Isola Structure

We now focus on the family of 9-reset cycles which exist in an interval of $I_s$ for which the 7- and 8-reset cycles are unstable during the transition from a 7- to an 8-reset cycle and *vice versa* (see Fig. 2b).

**Fig. 7:**
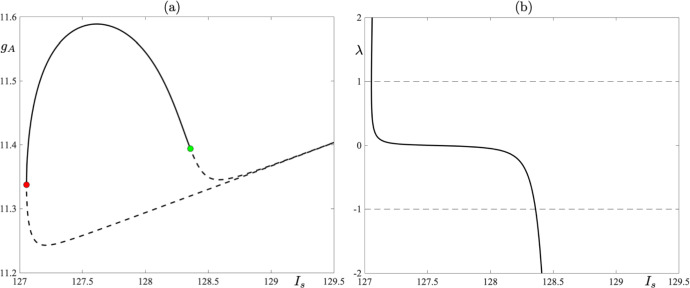
**a** One-parameter bifurcation diagram of system (1)–(2) with respect to parameter $I_s$. The bifurcation curve forms an isola with a fold point at $I_s \approx 127.0538$ (red dot). Stable branches are plotted as solid black lines, while unstable ones are plotted as dashed black lines. Also shown is a period-doubling bifurcation at $I_s\approx 128.358$ (green dot). **b** Floquet multipliers corresponding to the isola-like structure of the 9-reset cycles from panel (a) (Color figure online)

**Fig. 8:**
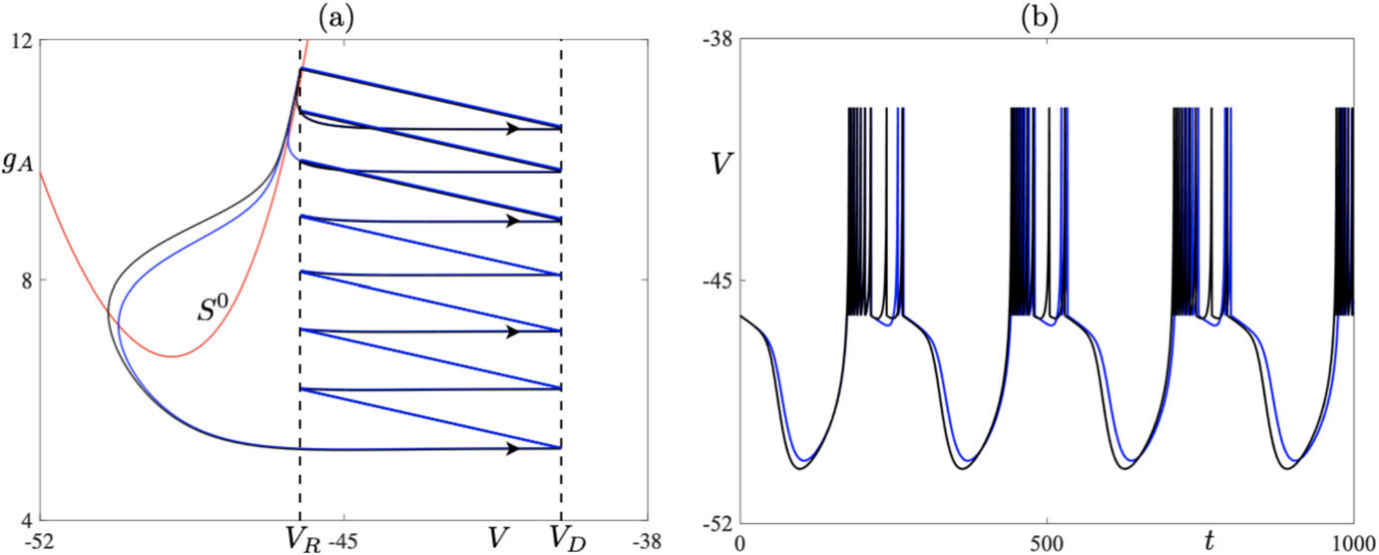
Two unstable 9-reset periodic solutions for $I_s = 131$ which form part of the isola-like structure depicted in Fig. 7: phase-plane projection (**a**) and membrane potential time profile (**b**) Note that, at resets, the initial conditions are close to each other for the two periodic cycles. Some of the segments are so close that without further zoom the presence of 9 resets is not discernible. Other parameter values are the same as in Fig. 6 (Color figure online)

Let us focus first on Fig. 7a. In the figure, we present a one-parameter bifurcation diagram of 9-reset cycles born at a fold bifurcation at $I_s\approx 127.0538$; see the red dot in the bifurcation diagram. By increasing $I_s$, we observe that the stable cycle loses its stability in a period-doubling bifurcation at $I_s \approx 128.4$; see the green dot in the bifurcation diagram. Corresponding values of the Floquet multiplier are depicted in Fig. 7b. Increasing $I_s$ further leads to a point on the bifurcation diagram, for $I_s\approx 129$, at which we seemingly observe that the two unstable cycles collide at another fold bifurcation, thus making the solution branch closed and creating an *isola of limit cycles* (Armbruster 1997). Numerical simulations show that we still have two distinct periodic cycles with canard segments, but after each reset the initial conditions of each cycle lie in a close proximity to each other; see Fig. 8 where two unstable 9-reset cycles for $I_s = 131$ are shown. The 9-reset cycle represented by the blue solid curve is characterised by the value of its non-trivial Floquet multiplier $\lambda = 318310$, and the black solid curve represents the 9-reset cycle with a nontrivial Floquet multiplier $\lambda ^* = -27 061$. Note that both $|\lambda |\gg 1$ and $|\lambda ^*|\gg 1$ with $|\lambda |\gg |\lambda ^*|$. Continuing with Fig. 8, we notice that the cycle represented by the blue curve is characterised by a longer trajectory segment along the repelling branch of the critical manifold, and hence the observed pronounced effect on the stability of the periodic solution.

Finally, numerical evidence suggests that the stable 7-reset and 9-reset cycles coexist in a small interval near $I_s\approx 127.2$. For $I_s \approx 128.4$, we observe the co-existence of the stable 8- and 9-reset cycles.

## Delayed Bursting

### The Effect of Canard Segments

**Fig. 9:**
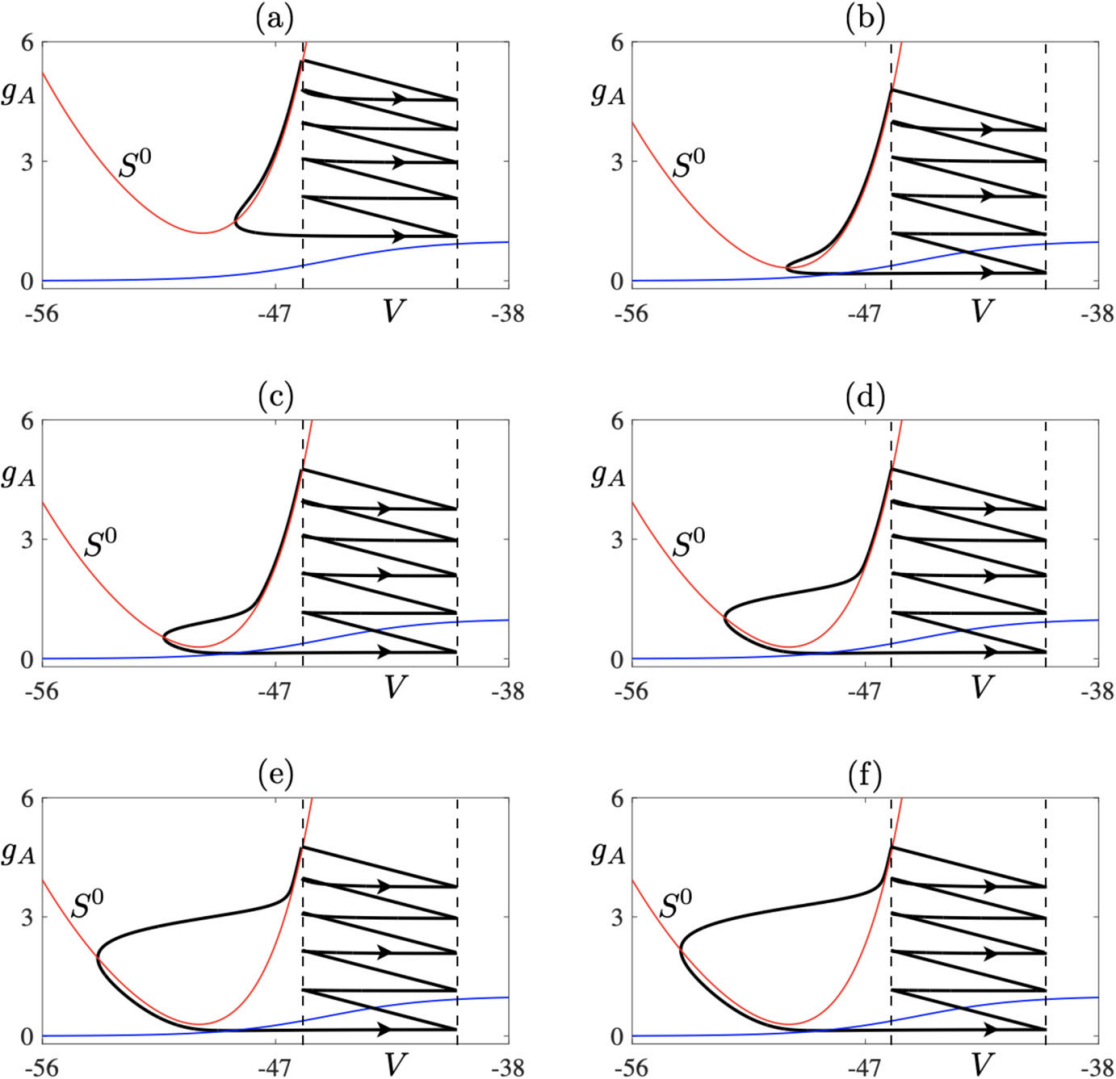
Unstable 5-reset canard cycle for $I_s = 120$ (**a**), $I_s = 102.5$ (**b**), $I_s = 101.8799$ (**c**), $I_s = 101.8614$ (**d**), $I_s = 101.8602$ (**e**), and with $I_s = 101.8554$ where the corresponding non-trivial Floquet multiplier is equal to $\lambda = -1.2441$ (**f**) (Color figure online)

**Fig. 10:**
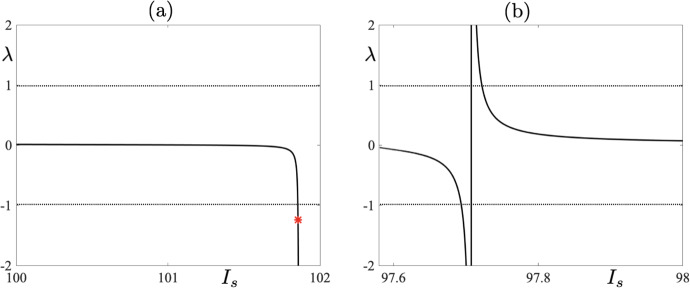
**a** Non-trivial Floquet multipliers for a family of 5-reset canard cycles corresponding to one-parameter bifurcation diagram shown in Fig. 11 for $I_s\in [100\,\,\, 102]$. The red star corresponds to limit cycle depicted in Fig. 9 in the lower right panel. **b** Same as in panel (a) but now for $I_s\in [97.5807,\,\, 98]$ (Color figure online)

We now focus on describing the dynamics of system (1)-(2) under the variation of $I_s$ in a regime termed as *delayed bursting* in Górski et al. (2021). (See Table 1 and this heading for the parameter values other than the bifurcation parameter $I_s$). Based on our numerical results and our previous theoretical work ( Desroches et al. 2021), we will argue that this dynamics is organised by three key elements: a fold point on the critical manifold, canard segments and the resets. These are showcased in Fig. 9. In panel (a), we present a 5-reset canard cycle for $I_s = 120$; other parameters are set to those given in Table 1 under the heading *delayed bursting*. This limit cycle is very unstable with a nontrivial Floquet multiplier $|\lambda |\gg 1$ due to the presence of a long canard segment, which one clearly sees lying along the $S^0_R$ (red parabola). Decreasing parameter $I_s$ has a twofold effect. Namely, it moves the *V*- and $g_A$-nullclines closer together ($g_A$-nullcline is depicted by the blue curve in Fig. 9), which in turns shortens the canard segment which lies along the repelling part of the *V*-nullcline. Thus, the value of the nontrivial Floquet multiplier decreases, and so does the period of the limit cycle. All the limit cycles depicted in Fig. 9 are unstable. Note that decreasing $I_s$ from $I_s = 120$ to $I_s = 102.5$ (panel (b)) leads to the nullclines coming closer together without having much of an effect on the length of the canard segment. Thus, in this parameter range, the effect of the variation of $I_s$ parameter is mostly on increasing the duration of the period of the 5-reset cycle. Consider now parameter variation in the range between $I_s = 101.8799$ and $I_s = 101.8554$ (panels (c–f)). In spite of the fact that this variation is of $O(10^{-2})$, the nontrivial Floquet multiplier changes by many orders of magnitude, being of order 1 for $I_s = 101.8554$. Thus, even though this parameter variation is rather small, it strongly influences the dynamics due to its effect on the stability. Looking at the figures, we notice that this is correlated to the changes in the length of the canard segment. Further decrease of parameter $I_s$ leads to the 5-reset cycle becoming stable, as shown in Fig. 10a depicting the values of the Floquet multiplier.

**Fig. 11:**
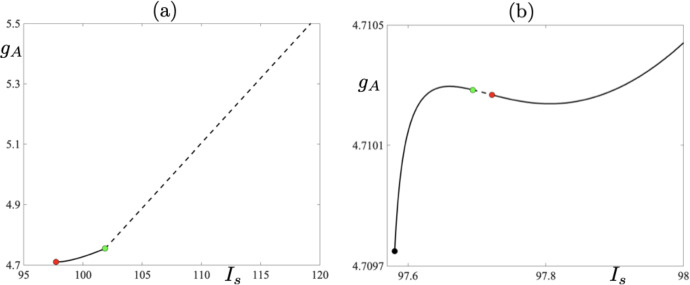
**a** One-parameter bifurcation diagram for $I_s\in [97.6028,\,\, 120]$. The red dot corresponds to the unstable limit cycle depicted in Fig. 9(f) - in the lower right panel - just “after” period-doubling bifurcation. **b** Continuation of the diagram for $I_s\in [97.5807,\,\, 98]$ down to the point of the homoclinic bifurcation (Color figure online)

Looking at Fig. 10a, we clearly see that the unstable 5-reset cycle (red star) gains stability and remains stable for $I_s < 101.84$. Moreover, looking at panel (b), where the notrivial Floquet multiplier is shown, reveals that there is a small parameter interval near $I_s\approx 97.7$, where the 5-reset cycle loses stability again. This is a subtle situation which, basically, corresponds to the crossing of the $g_A$-nullcline by the limit cycle trajectory below and to the left or right of the fold point of the critical manifold, depending on the parameter value. Hence, we observe the effect of the critical manifold’s fold point on the stability of the 5-reset cycle occurring under a very small parameter variation. We now link changes in the value of the nontrivial Floquet multiplier, which we described above, and the effect of canard segments as well as the presence of the critical manifold’s fold point, with the one-parameter bifurcation diagram depicted in Fig. 11a. It is plain to see that this bifurcation diagram has a piecewise character within the chosen range of $I_s$ values. We compare now this bifurcation diagram with the values of the corresponding Floquet multiplier depicted in Fig. 10a. We observe that at the point where the solution branch seems to become piecewise there occurs a sudden change in the value of the nontrivial Floquet multiplier of the 5-reset cycle. Note that, if this solution branch was truly piecewise, then we would observe a discontinuous jump of the corresponding Floquet multiplier precisely where the solution branch looses smoothness. We do not observe such a discontinuity in the values of the Floquet multiplier, but rather a sudden change of its value, which in the parameter range of the bifurcation diagram manifests itself in the piecewise-like behaviour of the solution branch. Note also that that the sudden change in the value of the Floquet multiplier occurs where the solution undergoes a period-doubling bifurcation; see the green dot in the bifurcation diagram and the green star in Fig. 10a correspoding the Floquet multiplier of the unstable 5-reset cycle close to the period-doubling bifurcation.

If we now focus on panel (b) of Fig. 10, we can see that there is yet another sudden change in the values of the Floquet multiplier near $I_s\approx 97.63$. This is due to the fact that when $I_s$ is varied within a small interval of $I_s\approx 97.63$, there the Floquet multiplier changes rapidly and the corresponding limit cycle has a short canard segment with the following characteristics: (a) when the multiplier is negative the canard segment surrounds the fold point, and (b) when the multiplier is positive the short canard segment is to the right of the fold point (numerical evidence not shown). Compare now the values of the Floquet multipler with the bifurcation diagram in Fig. 11b where the unstable 5-reset cycles are denoted by the dashed short segment. The green dot corresponds to a period-doubling bifurcation of the 5-reset cycle and the red one to a fold bifurcation on this branch.

### Homoclinic Bifurcation

**Fig. 12:**
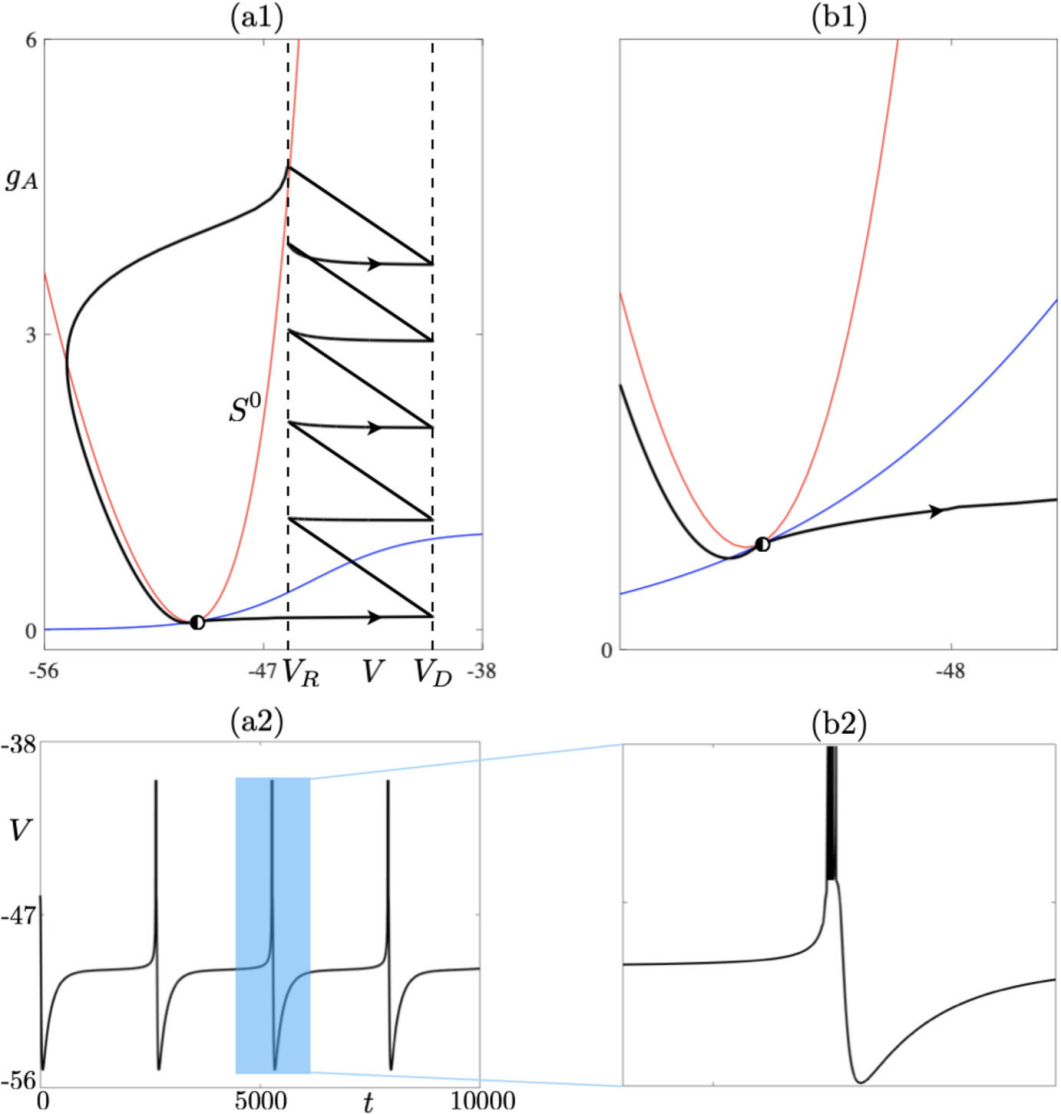
**a** 5-reset homoclinic connection for $I_s = 97.5807$, at the (putative) nonsmooth SNIC bifurcation. **a1** Phase-plane projection; **b1** zoom into the area where the two nullclines collide tangentially and create a saddle-node equilibrium point (half-filed disk). **a2** Membrane potential time profile; (b2) zoom around one 5-reset burst. Other parameter values correspond to the *delayed bursting* case, see Table 1 (Color figure online)

We will now discuss further Figs. 11 and 10b. In particular, we want to determine which event terminates solution branch from the left, that is, for decreasing values of $I_s$. Firstly, we note that within the chosen parameter regime, decreasing the values of $I_s$ implies that the two nullclines approach each other. The relative change in the position of the two nullclines also affects the limit cycle solution in that the change of the relative distance of the segments making up a limit cycle solution, from the *V*-nullcline, leads to the presence of canard cycles. This is visible in the behaviour of the non-trivial Floquet multiplier shown in Fig. 10a, as explained in the previous section. Moreover, the periodic solutions are also affected by the presence of an equilibrium point, for nearby parameter values of $I_s$, which slows down the evolution considerably. Decreasing $I_s$ down to $I_s\approx 97.5807$ makes the two nullclines meet tangentially. At the same time, the 5-reset cycle hits the equilibrium, which is born when the two nullclines collide, at $I_s\approx 97.5807$, thus undergoing a homoclinic bifurcation. The homoclinic connection terminating the family of 5-resets cycles is shown in Fig. 12 on the left panel; a zoom into the neighbourhood of the equilibrium point is in panel (b). The red parabola represents the critical manifold, and the blue curve the slow $g_A$-nullcline. Note the tangential interaction between the two nullclines and the limit cycle (black curve) which crosses the $g_A$-nullcline transversally and the *V*-nullcline tangentially. Finally, we should note that the value of the nontrivial Floquet multiplier converges to 0 at the homoclinic bifurcation due to the character of the equilibrium point, which is stable with respect to $g_A$ direction, and the resets induce stability in the *V* direction. This seems to suggest the presence of a saddle-node-on-an-invariant-circle (SNIC) bifurcation. Further investigation would be required to confirm it.

## The Effect of a Fold Point

**Fig. 13:**
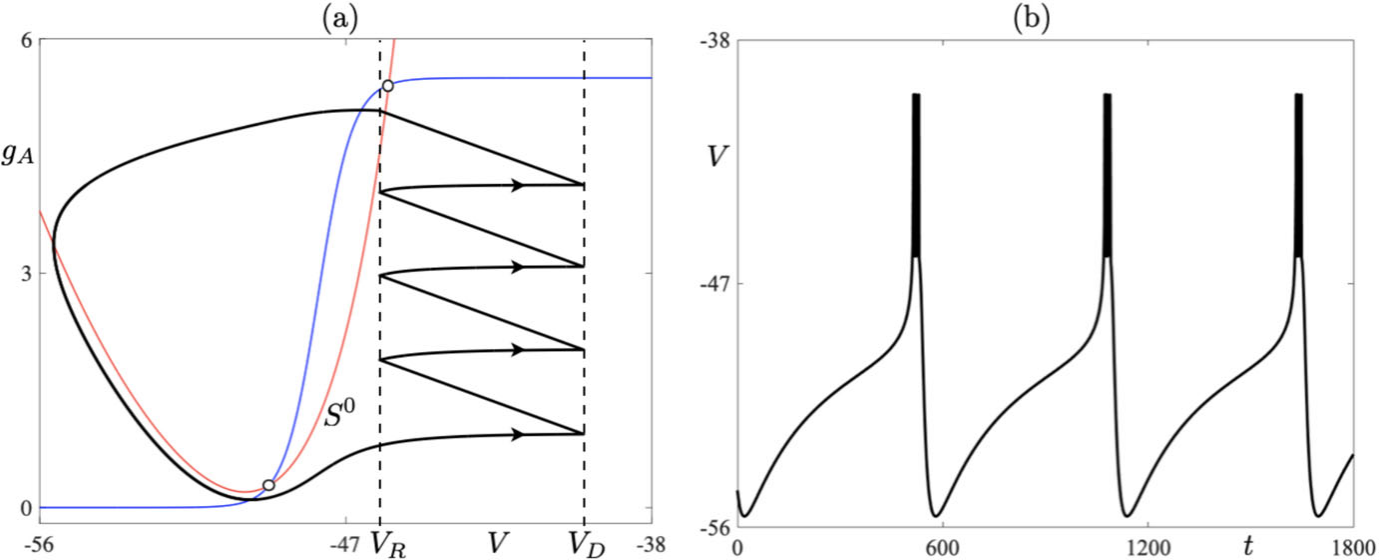
Effect of the fold of the critical manifold $S^0$ of system (1) on the delayed initiation of bursting on a 4-reset cycle obtained for $I_s=100$. This is due to the proximity of the nullclines near the fold point of $S^0$. Unstable equilibria are marked as open circles. **a** Phase-plane projection; **b** membrane potential time profile. All parameter values correspond to the *delayed bursting* case, see Table 1 (Color figure online)

There is a second mechanism in system (1)-(2) underpinning possible delayed activation of spikes or bursts. This is due to the shape of the critical manifold, which has a fold point. In the context of multiple-timescale dynamical systems, this geometry can cause a critical slowing-down of the dynamics when the slow nullcline (here the sigmoid-shaped nullcline of variable $g_A$) crosses transversally the fast nullcline (critical manifold) near its fold point. This is related to the canard phenomenon (Krupa and Szmolyan 2001; Dumortier and Roussarie 1996) and it has close links with type-2 excitability (Desroches et al. 2013; Wechselberger et al. 2013; De Maesschalck and Wechselberger 2015). Namely, trajectories slow down when passing near the fold point of the critical manifold $S^0$ and then follow the repelling part of $S^0$. In the context of periodic behaviour, this gives rise to canard cycles. Noteworthy, such canard cycles are of different type than the ones discussed earlier, for which the segment near the repelling part of $S^0$ initiates near, and is due to, the reset line. Figure 13 illustrates such a fold-induced canard cycle with 4 resets. Therefore, in a model of the type of CAdEx, there are two mechanisms inducing slowing-down of the dynamics before or after a spike: via a reset-induced canard segment and via a fold-induced canard segment.

## Conclusions

In the paper, we presented a numerical analysis of a spike-increment scenario in the CAdEx neuronal model introduced in Górski et al. (2021). We showed that under continuous parameter variation the spike increment follows a DIB, which we previously explained analytically in Desroches et al. (2021). We showed that the spike increment is induced by an interaction between a reset and a canard segment making up a periodic solution with resets. The scenario which we explained numerically occurs under continuous variation of parameter $I_s$ and the number of spikes changes from 7 to 8, with an intermediate 9-reset cycle. Moreover, this scenario was found to be accompanied by a fold bifurcation of the 8-reset cycle and a period-doubling bifurcation of the 7-reset cycle with a stable 9-reset cycle existing for parameter values between the fold and period-doubling bifurcations. The 9-reset cycles form an isola structure in parameter space. We also report the effect of canard segments on the 9-reset cycles. We can see that the stability of canard cycles changes by many orders of magnitude under exponentially-small parameter variation, which is a hallmark of canard-explosive transitions. It should be noted that, as we reported in Desroches et al. (2021), in the spike-increment scenario the 7-, 8- and 9-reset cycles do not connect in parameter space, instead there exist gaps between the corresponding bifurcation curves.

We also reported a homoclinic bifurcation of a 5-reset canard cycle. We show how the canard cycles and their stability change under exponentially-small parameter variation. In particular, we describe the effects of the fold point on the bifurcation diagram and the Floquet multiplier. A similar scenario was reported in our work in Desroches et al. (2021). The difference is that there we did not have a fold, but the stable and unstable parts of the critical manifold met in a corner. Note that the scenarios studied here, albeit with a specific number of resets, remain valid for other numbers of reset. We describe mechanisms and analyse organising centres that control in general the dynamical behaviour of system (1)-(2) and how its attractors change oscillatory patterns upon parameter variation.

From the neural modelling perspective, we should note that the two cases we have just described refer to what in Górski et al. (2021) is termed as *adaptive spiking* and *delayed bursting*, respectively. In particular, the first scenario of spike increment explains the *adaptive spiking* dynamics, whereas the second of these explains the *delayed bursting* scenario. *Delayed bursting* is characterised by a long period of quiescence, which is triggered by a dynamical phase close to the fold point of the critical manifold. More precisely, under parameter variation the *V*- and $g_A$- nullclines approach each other, and at the putative SNIC bifurcation they connect tangentially. The 5-reset periodic solution shown in Fig. 12 contains segments of trajectories evolving between the two nullclines, which for parameter values close to the SNIC bifurcation implies a significant slowing down of system’s evolution. This is the reason why in Górski et al. (2021), for parameter values indicated as *Delayed bursting* (see Table 1), the authors observe long quiescence periods after spiking.

More generally, DIB and reset-induced canards are expected in any IF model with a repelling slow manifold that can intersect the reset curve. In particular, this is for sure the case of the Izhikevich model (Izhikevich 2003); this is also the case of adaptive IF model such as those studied in Coombes et al. (2012) and in Desroches et al. (2021); and this is of course the case of the AdEx on top of the CAdEx studied here. Hence, this covers a number of key examples of IF model amongst the most popular and the most recently studied ones.

In terms of biological relevance, the specific solutions studied in the present work based on our previous results (Desroches et al. 2021), associated with DIB and reset-induced canard segments, are organising the transitions between different oscillatory patterns. These patterns and the associated signature carry pertinent information about the underlying signals, therefore the transition solutions (i.e., reset-induced canards), acting as thresholds between different signatures, are important even in terms of neural activity. Establishing a more precise link with neural coding goes beyond the scope of the present work and is certainly an interesting question for future work.

Finally, modeling neuronal activity with discontinuities is a way to account for slow-fast transitions (fast becoming instantaneous). IF models have been introduced to simplify biophysical models, in link with large-scale computations in large neuronal networks, as well as, establishing mean-field limits of such networks, while keeping enough biological details so that the model avoids becoming a mere toy. Such DIB and reset-induced bifurcations are the manifestation of the discontinuities but they have counterparts in smooth models, namely, spike-adding transitions (see, e.g., Desroches et al. 2022), which are purely due to the geometry of these biophysical models and the geometry of excitability (Izhikevich 2007).

## Data Availability

The authors declare that there are no data associated with this work.

## Nondimensionalisation

It may prove useful for analytical and numerical purposes to analyse system (1) in a nondimensional form. Therefore, we now perform a nondimensionalisation procedure of the model. In spite of the presence of resets, the nondimensionalisation may be applied and standard similarities between dimensional and nondimensional equations will be preserved. We note here that the main purpose of introducing the nondimensional version of the system is to explicitly show the time scale separation between the conductance and voltage variables. We show it explicitly by giving a numerical value of $p_0$ (defined below) in one representative case of the dynamic behaviour—namely adaptive spiking. It is straightforward to check other cases.

We introduce the following rescaling of the time and of the state variables:

A1 $$\begin{aligned} U = - \frac{V}{E_A},\quad \tau = \frac{t}{\tau _A},\quad g = \frac{g_A}{\bar{g}_A}. \end{aligned}$$

We then have:

$$ \frac{dV}{dt} = \frac{d V}{dU}\frac{d U}{d\tau }\frac{d\tau }{dt} = -E_A\frac{d U}{d\tau }\frac{1}{\tau _A} = -\frac{E_A}{\tau _A}\frac{d U}{d\tau }, $$

and the voltage equation takes the form:

A2 $$\begin{aligned} \begin{aligned} \frac{d U}{d\tau }&= -\frac{\tau _Ag_L}{C_m}\left( \frac{E_L}{E_A} + U\right) - \frac{\tau _Ag_L\Delta _T}{E_AC_m}\exp \left( -\left( U + \frac{V_T}{E_A}\right) \frac{E_A}{ \Delta _T}\right) ... \\&\quad - \frac{\tau _A\bar{g}_A}{C_m}g(1 + U) - \frac{\tau _AI_s}{E_AC_m}. \end{aligned} \end{aligned}$$

Now let:

$$ \frac{\tau _Ag_L}{C_m} = p_0 $$

Its inverse may be thought off as a small parameter $\varepsilon$. (A numerical example is given in section B). For the brevity of notation, we set:

A3 $$\begin{aligned} \begin{aligned} p_1&= \frac{E_L}{E_A},\quad p_2 = \frac{\tau _Ag_L\Delta _T}{E_AC_m},\quad p_3 = \frac{E_A}{\displaystyle \Delta _T},\\ p_4&= \frac{V_T}{E_A},\quad p_5 = \frac{\tau _A\bar{g}_A}{C_m},\quad \quad p_6 = \frac{\tau _AI_s}{E_AC_m}. \end{aligned} \end{aligned}$$

The voltage equation (A2) can now be written as:

A4 $$\begin{aligned} \frac{d U}{d\tau } = -p_0(p_1 + U) - p_5g(1 + U) - p_2\text{ e}^{\displaystyle (-p_3(U + p_4) )} - p_6. \end{aligned}$$

The conductance equation has the form:

$$ \frac{dg}{d\tau } = \frac{1}{1+ \exp \left( \frac{\displaystyle V_A}{\displaystyle \Delta _A} + \frac{\displaystyle E_A}{\displaystyle \Delta _A} U\right) } - g. $$

Again, for the brevity of notation, by setting:

$$ p_7 = \frac{V_A}{\Delta _A},\quad p_8 = \frac{E_A}{\Delta _A}, $$

we can rewrite the conductance equation as:

A5 $$\begin{aligned} \frac{dg}{d\tau } = \frac{1}{1+ \exp \left( p_7 + p_8 U\right) } - g. \end{aligned}$$

Writing (A4) and (A5) together, we thus have

A6 $$\begin{aligned} \begin{aligned} \varepsilon \frac{d U}{d\tau }&= -(p_1 + U) - \tilde{p}_5g(1 + U) - \tilde{p}_2\text{ e}^{-p_3(U + p_4)} - \tilde{p}_6,\\ \frac{dg}{d\tau }&= \frac{1}{1+ \exp \left( p_7 + p_8 U\right) } - g, \end{aligned} \end{aligned}$$

with: $\varepsilon =1/p_0$, $\tilde{p}_5=\bar{g}_A/g_L$, $\tilde{p}_2=\Delta _T/E_A$, $\tilde{p}_6=I_s/(g_LE_A)$. For completeness, applying nondimensionalisation to the reset condition provides us with the following reset rule:

A7 $$\begin{aligned} \text{ If }\quad U \ge -\frac{\displaystyle V_D}{\displaystyle E_A}\quad \text{ then }\quad {\left\{ \begin{array}{ll}U\mapsto -\frac{V_R}{E_A},\\[0.1cm] g\mapsto g + \frac{\displaystyle \delta g_A}{\displaystyle \bar{g}_A}. \end{array}\right. } \end{aligned}$$

We now introduce three additional nondimensional parameters:

$$ p_9 = -\frac{\displaystyle V_D}{\displaystyle E_A},\quad p_{10} = -\frac{V_R}{E_A}, \quad p_{11} = \frac{\displaystyle \delta g_A}{\displaystyle \bar{g}_A}. $$

## Parameter Values for Distinct Types of Spiking Patterns

In Table 1, we present parameter values which we use as initial set for the continuation runs presented in the current work; these values were used in Górski et al. (2021). Following the aforementioned work we classify each parameter set with the label description given in this work. If we choose one of the examples in the table, we can see that, in the case of adaptive spiking,

$$ p_0 = 10 = \frac{1}{\varepsilon }, $$

and so the time separation between the conductance and voltage variables is of the order of 10.

**Table 1:** Numerical values of parameters used in Table 1 in Górski et al. (2021)

	$C_m$	$E_A$	$E_L$	$I_s$	$V_A$	$\Delta _A$	$V_R$	$V_T$	$\delta g_A$	$\bar{g}_A$	$g_L$	$\tau _A$	$\Delta _T$	$V_D$
Adaptive spiking	200	−70	-60	200	−50	5	−55	−50	1	10	10	200	2	−40
Tonic spiking	200	−70	−70	192	−45	5	−56	−50	0	2	10	40	2	−40
Bursting	200	−60	−58	150	−45	1	−46	−50	1	10	10	200	2	−40
*Delayed bursting*	200	−70	−60	100	−45	2	−46	−50	1	1	12	100	2	−40
Accelerated spiking	200	−70	−60	130	−60	−5	−58	−48	0	6	10	300	2	−40
Chaotic-like spiking	200	−70	−58	90	−40	5	−47	−50	1	10	10	25	2	−40
